# Modulation of podocyte extracellular matrix remodeling in membranous nephropathy by the NFATc3/LRRC55/BK channel pathway

**DOI:** 10.1002/ccs3.70022

**Published:** 2025-06-13

**Authors:** Yaling Guo, Jingliang Min, Baochao Chang, Lei Liu, Jiqiang Zhang, Weidong Chen

**Affiliations:** ^1^ Department of Nephrology The First Affiliated Hospital of Jinan University Guangzhou Guangdong China; ^2^ Department of Nephrology The First Affiliated Hospital of Bengbu Medical University Bengbu China; ^3^ Department of Neurosurgery The Second Affiliated Hospital of Bengbu Medical University Bengbu China

**Keywords:** BK channel, extracellular matrix remodeling, LRRC55, membranous nephropathy, NFATc3, podocyte injury

## Abstract

Membranous nephropathy (MN) is a common glomerular disease characterized by podocyte injury. Although previous studies highlighted the leucine‐rich repeat‐containing 55/big potassium (LRRC55/BK) channel axis in Ang II‐induced apoptosis, our study further investigates the upstream regulation by nuclear factor of activated T‐cells 3 (NFATc3) and its role in extracellular matrix (ECM) remodeling. Using an Ang II‐induced podocyte injury model, we found that NFATc3 overexpression promoted LRRC55 transcription, increased BK channel activity, and elevated intracellular calcium, thereby exacerbating podocyte apoptosis and impairing migration. RNA‐seq and functional assays revealed significant upregulation of ECM‐related genes, with enhanced fibronectin and collagen I deposition. Patch‐clamp experiments confirmed BK channel activation was LRRC55‐dependent. In vivo, NFATc3 knockdown attenuated renal injury, restored podocyte markers (nephrin, WT1, synaptopodin), and alleviated proteinuria and fibrosis, whereas LRRC55 overexpression or BK agonist NS1619 reversed these effects. These findings reveal that NFATc3 aggravates Ang II‐induced podocyte injury through transcriptional regulation of LRRC55 and activation of the BK channel, contributing to ECM remodeling and glomerular dysfunction. Our results offer mechanistic insight into MN progression and suggest the NFATc3/LRRC55/BK axis as a potential therapeutic target.

## Graphical abstract

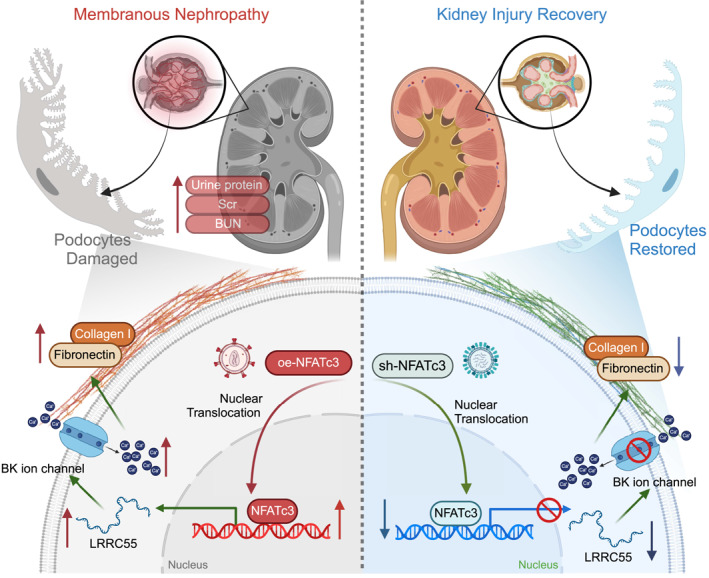

## 1 INTRODUCTION

Membranous nephropathy (MN) is a chronic glomerular disease primarily characterized by the deposition of immune complexes.^1^ Pathologically, it manifests as thickening of the glomerular basement membrane (GBM), accompanied by podocyte injury.^2^, ^3^ The most common clinical symptom of MN is significant proteinuria, typically resulting from podocyte damage.^4^ Podocytes play a crucial role in the glomerular filtration barrier; their impairment not only affects renal filtration function but may also lead to renal fibrosis and chronic renal failure.^5^, ^6^, ^7^ Studies have shown that podocyte injury is closely associated with renal inflammatory responses, extracellular matrix (ECM) remodeling, and cellular migration, proliferation, and apoptosis.^8^, ^9^, ^10^, ^11^, ^12^, ^13^, ^14^ In the pathogenesis of MN, angiotensin II (Ang II) is recognized as a critical regulator, increasingly drawing research attention. Ang II modulates podocyte function and survival by activating multiple signaling pathways, particularly under pathological conditions, where its role is more pronounced.^15^, ^16^, ^17^ A deeper understanding of MN facilitates the elucidation of the mechanisms underlying podocyte injury, thereby providing new targets and strategies for clinical intervention.

The nuclear factor of activated T‐cells (NFAT) transcription factor family plays a pivotal role in the activation, proliferation, differentiation, and apoptosis of immune cells. Recent studies have shown that the nuclear factor of activated T‐cells 3 (NFATc3) not only participates in immune responses but also exerts significant effects in nonimmune cells, particularly in renal‐associated diseases.^18^, ^19^ NFATc3 modulates inflammatory responses, ECM remodeling, and cellular proliferation and migration in renal cells by regulating gene expression.^19^, ^20^, ^21^, ^22^ Research on podocyte injury has revealed that NFATc3 enhances the cellular response to external damage through interactions with multiple signaling pathways. For instance, NFATc3 interacts with glycogen synthase kinase‐3 beta (GSK‐3β) and AMP‐activated protein kinase to regulate cellular metabolism and function.^23^, ^24^, ^25^ Furthermore, the activation of NFATc3 is closely linked to podocyte injury induced by Ang II, highlighting its potential role in MN. Studies indicate that inhibiting NFATc3 can alleviate podocyte damage,^26^ making it a significant target for research.

Leucine‐rich repeat‐containing 55 (LRRC55) is a newly discovered cell membrane protein that plays a regulatory role across various cell types. LRRC55 modulates intracellular calcium concentrations through interactions with potassium ion channels, thereby influencing cell proliferation, migration, and apoptosis.^27^ In the kidney, particularly within podocytes, LRRC55's expression is closely associated with renal cell functionality, and it is considered to play a critical role in the development of kidney injury.^28^ The big potassium (BK) channel, associated with LRRC55, regulates membrane potential and intracellular ionic balance as a potassium ion channel. In podocytes, activation of the BK channel alters the cells' electrophysiological properties, affecting their function and injury response.^28^, ^29^, ^30^, ^31^ Studies have found that LRRC55 regulates the activity of BK channels, contributing to the regulation of ECM remodeling in podocytes.^28^, ^32^ This process is potentially crucial in the progression of MN, although the exact mechanisms remain to be fully elucidated. Therefore, exploring the relationship between LRRC55 and the BK channel offers a promising research direction for the treatment of MN.

This study aims to elucidate the molecular mechanisms by which NFATc3/LRRC55 modulates ECM remodeling through BK ion channels, thereby exacerbating podocyte damage induced by Ang II in MN. Initially, we established a model of podocyte injury induced by Ang II to investigate the role of NFATc3, employing RNA‐seq technology to identify key molecules involved in ECM remodeling and to analyze their mechanisms of action. Subsequently, we evaluated the interrelationship between NFATc3 and the LRRC55/BK channel axis in podocyte damage, revealing their potential roles in MN. Lastly, by modulating NFATc3 expression, we assessed its effects on podocyte migration, proliferation, and apoptosis, thereby clarifying the function of NFATc3 in MN.

Not only does this research contribute to unveiling the molecular mechanisms of podocyte damage in MN, but it also provides new insights into the synergistic effects of NFATc3/LRRC55/BK channels in the pathophysiology of renal diseases. Scientifically, exploring these mechanisms offers new targets for the prevention and treatment of renal diseases, particularly in preventing podocyte damage and ECM remodeling, which have significant clinical implications. Furthermore, targeted interventions against the NFATc3/LRRC55/BK channel axis may represent novel therapeutic approaches for MN and other related renal diseases, enhancing the quality of life for patients with renal diseases and slowing the progression of renal failure.

## 2 MATERIALS AND METHODS

### 2.1 RNA extraction and sequencing

The podocytes from the control group and the experimental group with model‐induced kidney injury were collected, with 8 samples in each group. Total RNA was isolated using TRIzol reagent (15596026, Invitrogen). The concentration and purity of RNA samples were measured using a NanoDrop 2000 spectrophotometer (1011U, NanoDrop). Total RNA samples meeting the following criteria were used for subsequent experiments: RNA integrity number ≥7.0, and 28S:18S ratio ≥1.5.

The sequencing libraries were prepared and sequenced by CapitalBio Technology. A total of 5 μg of RNA per sample was used. Briefly, ribosomal RNA was removed from total RNA using the Ribo‐Zero™ Magnetic Kit (MRZE706, Epicentre Technologies). The NEBNext Ultra RNA Library Prep Kit for Illumina (#E7775, NEB) was used to construct sequencing libraries. RNA was fragmented into approximately 300 base pair segments in NEBNext First Strand Synthesis Reaction Buffer (5X). First‐strand cDNA was synthesized using reverse transcriptase primers and random primers, followed by second‐strand cDNA synthesis in dUTP Mix (10X). The cDNA fragments were end‐repaired, including the addition of a poly(A) tail and ligation of sequencing adapters. Following adapter ligation, the USER Enzyme (#M5508, NEB) was used to digest the second cDNA strand, constructing strand‐specific libraries. The library DNA was then amplified, purified, and enriched through PCR. The libraries were characterized using the Agilent 2100 system and quantified using the KAPA Library Quantification Kit (KK4844, KAPA Biosystems). Finally, paired‐end sequencing was performed on the NextSeq 500 system (Illumina).

### 2.2 Quality control and reference genome alignment of sequencing data

We employed FastQC software (v0.11.8) to assess the quality of paired‐end reads from raw sequencing data. The raw data were preprocessed using Cutadapt software (v1.18) to remove Illumina sequencing adapters and poly(A) tails. Reads containing more than 5% N bases were discarded using a Perl script. The FASTX‐Toolkit (v0.0.13) was utilized to select reads where 70% of bases had a quality score above 20. BBMap software was used for the repair of paired‐end sequences. Finally, high‐quality filtered reads were aligned to the mouse reference genome using HISAT2 software (v0.7.12).

### 2.3 Differential expression analysis

Gene expression data were normalized and quantitatively adjusted using the limma package (version 3.48.3) in R to mitigate batch effects and technical noise. Differential expression analysis was performed on the normalized data using DESeq2 (version 1.32.0) and edgeR (version 3.34.1), identifying significantly upregulated or downregulated mRNAs. Selection criteria were set at a *p*‐value <0.05 and an absolute log_2_ fold change >1.

### 2.4 Weighted gene co‐expression network analysis

To analyze gene expression profiles, we calculated the median absolute deviation (MAD) for each gene and excluded the bottom 50% with the lowest MAD values. Outlier genes and samples were further removed using the goodSamplesGenes function from the R package weighted gene co‐expression network analysis (WGCNA). A scale‐free co‐expression network was then constructed using WGCNA, with a minimum gene module size set to 50 and a soft‐thresholding power of 13. Modules with a correlation distance of <0.7 were merged, resulting in three co‐expression modules. Pearson correlation analysis (*p* < 0.05) was performed to assess the association between the modules and experimental groups. The module most significantly associated with Ang II treatment was identified as the disease‐related gene module and selected for subsequent analysis.

### 2.5 LASSO regression algorithm

In our bioinformatics study, we employed the least absolute shrinkage and selection operator (LASSO) regression method to identify key genes associated with disease. To ensure reproducibility, we first set a random seed and utilized the glmnet package to handle high‐dimensional datasets. Candidate genes were subjected to LASSO regression using the glmnet function, modeling the data as a binary classification problem. The class labels were extracted from sample names via regular expressions and used as response variables. Model evaluation was performed by plotting the model object and applying cross‐validation using cv.glmnet to determine the optimal lambda value. Finally, genes corresponding to nonzero coefficients at the optimal lambda value were identified as key genes associated with disease status and subsequently reported.

### 2.6 GO and KEGG enrichment analysis

Gene Ontology (GO) and Kyoto Encyclopedia of Genes and Genomes (KEGG) enrichment analyses were performed using the “clusterProfiler” package in R. Candidate target genes or disease‐related differentially expressed genes (DEGs) were selected based on a significance threshold of *p* < 0.05. GO analysis was conducted to categorize genes into three domains: biological process (BP), molecular function, and cellular component. This allowed the identification of key cellular functions and signaling pathways associated with DEGs, as well as their involvement in disease‐related pathways. KEGG enrichment analysis was performed using the “clusterProfiler” package in R, ranking pathways based on *p*‐values. The results were visualized through bubble plots to provide a comprehensive overview of the enriched pathways.

### 2.7 Construction of PPI networks

The STRING database (http://www.string‐db.org/) integrates experimental data, text mining results from PubMed abstracts, information from other curated databases, and computational predictions to study protein–protein interactions (PPIs). Using the STRING database, we analyzed the interactions of 12 key factors with a confidence score threshold of 0.15. Subsequently, R software was employed to quantify and visualize the number of protein connection nodes. The greater the number of connection nodes, the higher the centrality of a protein within the network.

### 2.8 Lentiviral transfection

To achieve gene overexpression, the plasmid vector pCMV6‐AC‐GFP (LM‐2069, LMAI Bio) was used to construct NFATc3 or LRRC55 overexpression plasmids, which were synthesized by Sangon Biotech. The transfection series, based on the pLKO.1‐puro vector (QYV0024, QuaYad), is detailed in Table S1. Lentiviral particles for NFATc3 overexpression (oe‐NFATc3‐LTEP‐s, hereafter referred to as oe‐NFATc3), LRRC55 overexpression (oe‐LRRC55‐LTEP‐s, hereafter referred to as oe‐LRRC55), and NFATc3 knockdown (sh‐NFATc3‐LTEP‐s, hereafter referred to as sh‐NFATc3) were generated using HEK293T cells (CBP60661, COBIOER). The plasmids and lentiviral packaging services were provided by Sangon Biotech. To construct luciferase‐reporter lentiviral plasmids (oe‐NFATc3‐luc, oe‐LRRC55‐luc, sh‐NC‐luc, sh‐NFATc3‐luc, and sh‐LRRC55‐luc), the target plasmids were co‐transfected into HEK293T cells using Lipofectamine 2000 (11668030, Thermo Fisher Scientific), along with auxiliary plasmids. After verification, amplification, and purification, the packaged lentiviruses were obtained.

For lentivirus‐mediated cell transfection, 5 × 10^5^ cells were seeded in six‐well plates. When cell confluence reached 70%–90%, the culture medium containing packaged lentivirus (MOI = 10, with a working titer of approximately 5 × 10^6^ TU/mL) and 5 μg/mL polybrene (TR‐1003, Sigma‐Aldrich) was added. After 4 h, the medium was diluted with an equal volume of fresh culture medium to reduce polybrene toxicity. At 24 h post‐transfection, the culture medium was replaced with a fresh medium. At 48 h, transfection efficiency was assessed using the luciferase reporter gene. To establish a stable cell line, transfected cells were subjected to selection with 10 μg/mL puromycin (A1113803, Gibco). Once the puromycin‐resistant cells stabilized in culture, they were collected for further analysis. Gene overexpression and knockdown efficiency were validated using reverse transcription quantitative polymerase chain reaction (RT‐qPCR).^33^

### 2.9 Cell transfection and experimental grouping

MPC5 cells (CL‐0855, procell life science, China) podocytes were cultured in medium containing 10% fetal bovine serum (Gibco, Cat. No. A5256701) and 1% penicillin‐streptomycin (Gibco, Cat. No. 15140148) at 37°C in a 5% CO_2_ humidified incubator. Cells were maintained at 37°C in a 5% CO_2_ incubator. Once they reached 70%–90% confluence, transfection was performed using Lipofectamine 3000 (Invitrogen, Cat. No. L3000015). Depending on the experimental design, cells were transfected with either overexpression plasmids (oe‐NFATc3 or oe‐LRRC55) or shRNA plasmids (sh‐NFATc3 or sh‐LRRC55). Empty plasmids (oe‐NC) and scrambled shRNA (sh‐NC) served as controls.

After 48 h of transfection, all groups were treated with 10 μM Ang II (Sigma‐Aldrich, Cat. No. 05‐23‐0101) for 24 h to induce podocyte injury. In selected groups, the BK channel agonist NS1619 was added to conduct a rescue experiment. The experimental groups were as follows:

Control group: MPC5 cells cultured under normal conditions without any treatment. Ang II group: Cells treated with 10 μM Ang II for 24 h. Ang II + oe‐NC + sh‐NC group: Cells treated with Ang II and transfected with control plasmids (oe‐NC and sh‐NC). Ang II + oe‐NFATc3 group: Cells treated with Ang II and transfected with an NFATc3 overexpression plasmid. Ang II + sh‐NFATc3 group: Cells treated with Ang II and transfected with an NFATc3 shRNA plasmid. Ang II + oe‐LRRC55 group: Cells treated with Ang II and transfected with an LRRC55 overexpression plasmid. Ang II + sh‐LRRC55 group: Cells treated with Ang II and transfected with an LRRC55 shRNA plasmid. Ang II + sh‐LRRC55 + NS1619 group (rescue group): Cells treated with Ang II and transfected with an LRRC55 shRNA plasmid, followed by the addition of the BK channel agonist NS1619. These experimental conditions were designed to investigate the role of NFATc3 and LRRC55 in Ang II‐induced podocyte injury and ECM remodeling.^26^

### 2.10 CCK‐8 assay

To evaluate the effects of Ang II treatment, NFATc3, and LRRC55 on the viability and proliferative capacity of podocytes, a cell counting kit‐8 (CCK‐8) assay (Sigma‐Aldrich, 96992) was performed. Treated podocytes were seeded at a density of 5 × 10^3^ cells per well in a 96‐well plate and incubated for 24 h. Subsequently, 10 μL of CCK‐8 solution was added to each well, followed by a 2‐h incubation at 37°C. The optical density (OD) was then measured at 450 nm using a microplate reader (iMark™ Microplate Absorbance Reader, Bio‐Rad). The changes in OD values were used to assess variations in cell proliferation and viability. The experiment was repeated three times, and the data are presented as the mean ± standard deviation (SD), ensuring reliable evaluation of the impact of different treatments on podocyte viability and proliferation.

### 2.11 Transwell migration assay

To evaluate the effect of NFATc3 on podocyte migration, a Transwell migration assay was performed using Transwell chambers with an 8‐μm pore size (#3422, Corning). Podocyte cells (2 × 10^4^ cells/well) were suspended in serum‐free dulbecco’s modified eagle medium (#11965092, Gibco) and seeded in the upper chamber. The lower chamber was filled with a complete medium containing 10% fetal bovine serum (FBS, #10099‐141, Gibco) to serve as a chemoattractant. After incubation at 37°C in a 5% CO_2_ humidified incubator for 24 h, nonmigrated cells on the upper side of the membrane were gently removed using a cotton swab. The migrated cells were then fixed with 4% paraformaldehyde (#P0099, Beyotime) for 15 min and stained with 0.1% crystal violet (#C0121, Beyotime) for 15 min. Following staining, the chambers were washed three times with phosphate‐buffered saline (PBS) to remove any nonspecific staining. The number of migrated cells was visualized and counted under an inverted microscope (ECLIPSE Ts2, Nikon). Cell quantification was performed using ImageJ software, and statistical analysis was conducted using GraphPad Prism 8.0 to determine the mean number of migrated cells.

### 2.12 Flow cytometry analysis

To assess podocyte apoptosis, annexin V‐FITC/PI dual staining was performed in conjunction with flow cytometry. Podocyte cells were treated, washed with PBS, and incubated with annexin V‐FITC and PI dyes (BD Biosciences, BMS500FI‐300) in the dark for 15 min. After incubation, apoptotic cells were analyzed using a BD FACSAria III flow cytometer (BD Biosciences). The collected data were processed using FlowJo software to quantify and visualize the percentage of apoptotic cells.

### 2.13 Patch‐clamp experiment

To assess BK channel activity, the whole‐cell patch‐clamp technique was employed. Podocyte cells were seeded onto sterile glass coverslips and cultured until they adhered adequately for experimentation. BK channel currents were recorded using an Axopatch 200B amplifier (Molecular Devices) and acquired via the Digidata 1440A Data Acquisition System (Molecular Devices). The recording solution contained 140 mM KCl and 10 mM 4‐(2‐hydroxyethyl)‐1‐piperazineethanesulfonic acid to maintain a stable potassium ion environment. Instantaneous current (I)–voltage (V) curves were recorded under varying voltage gradients to measure current density (pA/pF). Data were analyzed using pCLAMP 10.7 software (Molecular Devices). All experiments were conducted at room temperature (22–25°C) to ensure signal stability and reproducibility.

### 2.14 RT‐qPCR assay

Total RNA was extracted from cells or tissues using the TRIzol reagent kit (10296010, Invitrogen, Thermo Fisher Scientific). RNA quality and concentration were assessed using an ultraviolet‐visible spectrophotometer (ND‐1000, NanoDrop, Thermo Fisher Scientific). Reverse transcription was performed using the PrimeScript™ RT‐qPCR kit (RR086A, Takara). RT‐qPCR was conducted using the SYBR Premix Ex Taq™ (DRR820A, Takara) on a LightCycler 480 system (Roche Diagnostics). glyceraldehyde‐3‐phosphate dehydrogenase (GAPDH) was used as the internal control for mRNA normalization. The primers used for amplification were designed and synthesized by Shanghai General Biological Technology Co., Ltd., with sequences listed in Table S2. Gene expression levels were quantified using the 2^−ΔΔCt^ method, representing the fold‐change in target gene expression between the experimental and control groups. The ΔΔCt value was calculated as follows: ΔΔCt = ΔCt (experimental group) − ΔCt (control group), where ΔCt = Ct (target gene) − Ct (internal reference gene).

### 2.15 WB analysis

Cells and kidney tissue were collected and digested using trypsin (T4799‐5G, Sigma‐Aldrich). The collected cells and tissues were lysed with enhanced radioimmunoprecipitation assay buffer lysis buffer containing protease inhibitors (AR0108, Boster). Protein concentration was determined using a BCA Protein Assay Kit (AR1189, Boster).

Proteins were separated by sodium dodecyl sulfate–polyacrylamide gel electrophoresis and subsequently transferred onto polyvinylidene fluoride membranes. The membranes were blocked with 5% BSA (9048‐46‐8, Sigma‐Aldrich) at room temperature for 1 h, followed by incubation with diluted primary antibodies (detailed in Table S3) at 4°C overnight. After three washes with phosphate‐buffered saline with tween 20 (PBST) (3 × 5 min), the membranes were incubated with either anti‐rat HRP‐conjugated secondary antibody (31475, 1:5000; Thermo Fisher Scientific) or anti‐rabbit HRP‐conjugated secondary antibody (7074, 1:5000; Thermo Fisher Scientific) at room temperature for 1 h. The membranes were then washed three times with PBST (3 × 5 min). Following the removal of PBST, an appropriate amount of enhanced chemiluminescence (ECL) detection reagent (Omt‐01, OMIGET) was added, and the membranes were incubated at room temperature for 1 min. Excess ECL reagent was removed, and the membranes were sealed with plastic wrap. The membranes were exposed to x‐ray film in a darkroom for 5–10 min, followed by development and fixation. ImageJ software was used for densitometric quantification of Western blot (WB) bands, with β‐actin serving as the internal control.

### 2.16 ChIP assay

To investigate the binding of NFATc3 to the LRRC55 promoter, a chromatin immunoprecipitation (ChIP) assay was performed using the ChIP‐IT Express Magnetic ChIP Kit (Active Motif, 53008) in conjunction with an anti‐NFATc3 antibody. First, podocytes were cultured to 90% confluence and cross‐linked with 1% formaldehyde (Sigma‐Aldrich) for 10 min to preserve protein–DNA interactions. Cross‐linking was then quenched by adding 0.125 M glycine (Thermo Fisher Scientific). The cells were lysed, and chromatin was sheared via ultrasonication to obtain DNA fragments. The samples were incubated overnight with an NFATc3‐specific antibody (Thermo Fisher Scientific, PA5‐79734), followed by binding with protein A/G magnetic beads (Thermo Fisher Scientific). After bead washing, the immunoprecipitated complexes were collected, cross‐links were reversed, and DNA was extracted. Finally, DNA gel electrophoresis was used to assess the enrichment of NFATc3 at the LRRC55 promoter binding region, with normalization to control values.

### 2.17 Luciferase reporter gene assay

To investigate the transcriptional activation of the LRRC55 promoter by NFATc3, we constructed a luciferase reporter plasmid containing NFATc3 binding sites within the LRRC55 promoter region. Podocytes were cultured in 10% FBS (Gibco) and co‐transfected with 0.1 μg of firefly luciferase reporter plasmid, 0.02 μg of Renilla luciferase plasmid, and 0.1 μg of NFATc3 overexpression plasmid (oe‐NFATc3) or empty vector (sh‐NC + oe‐NC plasmid) using Lipofectamine 3000 (Invitrogen). The firefly luciferase constructs included both the wild‐type LRRC55 promoter (WT‐promoter) and a mutant LRRC55 promoter (MUT‐Promoter). After 48 h, firefly and Renilla luciferase activities were measured using the Dual‐Luciferase Reporter Assay System (Promega, E1960). Renilla luciferase activity was used for normalization, and results were expressed as relative luciferase units, comparing experimental groups to controls.

### 2.18 Site‐directed mutagenesis experiment

To further validate the impact of NFATc3 binding sites on LRRC55 promoter activity, site‐directed mutagenesis primers were designed (Table S4), targeting two NFATc3 binding sites. A Q5 Site‐Directed Mutagenesis Kit (NEB) was used to construct luciferase reporter plasmids containing the two site‐specific mutations in the LRRC55 promoter.

### 2.19 Establishment of the MN mouse model and animal grouping

To establish the MN model, male SPF‐grade C57BL/6 mice aged 10–12 weeks (*n* = 10 per group; strain 213; purchased from Beijing Vital River Laboratory Animal Technology Co., Ltd.) were used. All animal procedures conformed to the Guidelines for the Care and Use of Laboratory Animals of our institution. Mice were housed under standard conditions with a 12‐h light/dark cycle, a temperature of 25 ± 2°C, and a relative humidity of 55 ± 5%, with free access to food and water. Animals were acclimatized to these conditions for 2–3 days prior to the experiment.

The MN model was induced using a classical dual‐factor stimulation approach: Antigenic immune stimulation: A single tail vein injection of anti‐Fx1A antiserum (0.4 mL/100 g body weight) was used to induce subepithelial deposition of immune complexes along the GBM, mimicking the autoimmune characteristics of MN. Pro‐injury stimulus by angiotensin II: An ALZET osmotic minipump (model 1002, Durect Corp.) was implanted subcutaneously to continuously infuse angiotensin II (A9525, Sigma‐Aldrich) at a rate of 1000 ng/kg/min, or an equal volume of saline, for 28 days. This was intended to enhance podocyte mechanical stress and calcium signaling activation, inducing typical renal dysfunction associated with MN.

Mice were randomly assigned to six groups: Control group: Healthy mice without any treatment. Model + sh‐NC + oe‐NC group: MN mice injected with control lentivirus (sh‐NC and oe‐NC). Model + sh‐NFATc3 group: MN mice receiving NFATc3 knockdown via lentiviral delivery. Model + sh‐NFATc3 + oe‐LRRC55 group: MN mice with NFATc3 knockdown and LRRC55 overexpression. Model + sh‐LRRC55 group: MN mice with LRRC55 knockdown. Model + sh‐LRRC55 + NS1619 group: MN mice with LRRC55 knockdown treated daily with the BK channel agonist NS1619 (10 mg/kg; Sigma‐Aldrich) via intraperitoneal injection.^32^ Lentivirus was injected via the tail vein on days 3 and 14 post‐model induction (MOI = 100) to mediate NFATc3 knockdown, LRRC55 overexpression, or LRRC55 knockdown. At the end of the experiment, serum samples were collected for biochemical analysis, and 24‐h urine samples were collected to determine urinary protein levels.^34^

### 2.20 Isolation of mouse glomeruli and podocytes

Glomeruli were manually isolated from mouse kidneys at 4°C under a stereomicroscope. Pink spherical glomeruli were collected into RNAlater solution for RNA extraction using the RNA Pico Kit, and RNA quality and concentration were verified by PCR. For some samples, approximately 50 glomerular cross‐sections per mouse were further isolated using a laser capture microdissection system (Leica). For bulk glomerular preparation, mice were anesthetized and perfused via the heart with magnetic bead solution. Kidneys were harvested, decapsulated, and minced into ∼1 mm^3^ pieces. The tissue was digested at 37°C for 15 min in a solution containing 300 U/mL collagenase I, 1 mg/mL protease E, and 50 U/mL DNase I. After digestion, the suspension was filtered through a 100 μm cell strainer and centrifuged. The pellet was washed with PBS three times and purified using magnetic separation. Isolated glomeruli were used for in situ electrophysiological recording and intracellular potassium ion concentration measurement. To isolate podocytes, glomeruli were digested at 37°C for 10 min using 0.025% trypsin or collagenase IV. The reaction was stopped, and the suspension was sequentially filtered through 70 and 40 μm strainers to obtain a single‐cell suspension. Cells were then centrifuged at 300*g* for 2 min and resuspended in PBS containing 0.1% bovine serum albumin (BSA) and 2 mM ethylenediaminetetraacetic acid (EDTA). Podocytes were enriched using Dynabeads conjugated with anti‐podocalyxin antibodies (FlowComp Flexi Kit, Invitrogen). Trypan blue staining showed a cell viability rate >90%.

### 2.21 Scr, BUN, and urinary protein analysis

Mouse tail vein blood was collected and transferred into EDTA‐containing centrifuge tubes. The tubes were gently inverted to ensure uniform dispersion of EDTA. The samples were then allowed to stand at 4°C for 15–30 min and centrifuged at 1200 × g for 10 min to obtain the supernatant serum. The serum samples were stored at −20°C for further analysis. Prior to use, samples were thawed and gently mixed to maintain stability. Serum creatinine (Scr) levels were measured using a creatinine assay kit (C011‐2‐1, Nanjing Jiancheng), and absorbance was recorded at 510 nm using a spectrophotometer. Blood urea nitrogen (BUN) levels were determined with a BUN assay kit (C013‐2‐1, Nanjing Jiancheng) using an enzyme‐linked immunosorbent assay method. Urine samples were collected 24 h after animal modeling, and urinary protein concentration was quantified using the Pierce™ BCA Protein Assay Kit (23225, Thermo Fisher Scientific).

### 2.22 Immunohistochemical staining

To assess the expression of podocyte markers nephrin, WT1, and synaptopodin in renal tissues, kidney samples were fixed in 4% paraformaldehyde, embedded in paraffin, and sectioned to a thickness of 5 μm. The tissue sections underwent antigen retrieval by autoclaving in citrate buffer (pH 6.0) or Tris‐EDTA buffer (pH 9.0) at 121°C for 20 min. To quench endogenous peroxidase activity, sections were incubated in methanol containing 3% H_2_O_2_ for 5 min. After blocking nonspecific antibody binding with 10% goat serum, the sections were incubated overnight at 4°C with primary antibodies, including anti‐nephrin (PA5‐20330, Thermo Fisher Scientific), anti‐WT1 (MA5‐32215, Thermo Fisher Scientific), and anti‐synaptopodin (ab259976, Abcam). Following PBS washes to remove unbound antibodies, sections were incubated with horseradish peroxidase (HRP)‐conjugated secondary antibodies at room temperature for 60 min. Chromogenic detection was performed using a DAB staining kit (ab64238, Abcam). Images were captured using an optical microscope (Olympus), and protein expression levels were analyzed using ImageJ software.

### 2.23 Immunofluorescence assay

To investigate the colocalization of LRRC55 and the BK channel α‐subunit in podocyte cells and glomeruli, immunofluorescence staining was performed. First, treated podocyte cells or kidney tissue sections were fixed. After blocking with a serum‐free protein block solution (Agilent), primary antibodies against LRRC55 (ab121412, Abcam) and KCNMA1 (APC‐151, Alomone Labs) were applied and incubated overnight at 4°C. Unbound antibodies were removed by washing with PBS, followed by incubation with Alexa Fluor 488‐ and Alexa Fluor 594‐conjugated secondary antibodies (Thermo Fisher Scientific; A‐11001 and A‐11012). Colocalization of proteins in podocyte cells was observed under an optical microscope (Olympus), and quantitative analysis was performed using ImageJ software.

### 2.24 H&E staining and Masson's trichrome staining

To assess pathological changes in renal tissue, paraffin‐embedded kidney sections were subjected to hematoxylin and eosin (H&E) staining (Thermo Fisher Scientific) and Masson's trichrome staining (Solarbio).

For H&E staining, kidney tissue sections were deparaffinized and rehydrated using a graded ethanol series (100%, 95%, 85%, and 75%). Following a brief wash in distilled water, the slides were incubated in hematoxylin solution for 4 min. Excess hematoxylin was removed by rinsing under running tap water for 10 min. The sections were then briefly incubated in 1% acid alcohol for 5 s, followed by another rinse under running water for 3 min. Subsequently, eosin counterstaining was performed for 8 min. The sections were then dehydrated using graded ethanol solutions (95%, 100%, and 100%), cleared in xylene, and mounted with Cytoseal 60 (Stephens Scientific). The GBM thickening and tubular atrophy were observed under a light microscope (Olympus), and images were analyzed using ImageJ software.

For Masson's trichrome staining, kidney tissue sections were deparaffinized and rehydrated using the same graded ethanol series. Following a wash in distilled water, the sections were incubated in iron hematoxylin working solution for 5 min, washed, and briefly incubated in acid alcohol solution for 5 s, followed by a 5‐min wash under running water. The sections were then stained in ponceau fuchsin solution for 8 min, rinsed with distilled water, and incubated in phosphotungstic acid for 1 min. Without further washing, the sections were directly transferred to an aniline blue solution for 20 s, briefly rinsed in distilled water, and fixed in 1% acetic acid for 1 min. After washing, the sections were rapidly dehydrated with 95% and 100% ethanol, cleared in xylene, and sealed with a resin‐based mounting medium. Blue‐stained areas indicated fibrosis, whereas red‐stained areas represented muscle fibers, cytoplasm, fibrin, keratin, and red blood cells. ImageJ software was used to quantify fibrosis. Each image was segmented into distinct color channels, and appropriate thresholds were applied uniformly across all images. For each group, one kidney was analyzed, with three sections selected for Masson staining, spaced 50 sections apart. The percentage of fibrosis was calculated as fibrotic area/total section area × 100%.

### 2.25 TEM analysis

To evaluate the impact of LRRC55 on the ultrastructure of podocytes, transmission electron microscopy (TEM) was employed to observe podocyte foot process fusion and cytoskeletal alterations. Processed podocytes and renal cortex tissue (cut into small fragments <1 mm^2^) were fixed at 4°C with 2.5% glutaraldehyde (G5882, Sigma‐Aldrich). Tissue samples were washed with 0.2 mol/L PBS and incubated with 1% osmium tetroxide (75632, Sigma‐Aldrich) at room temperature for 2 h. Following ethanol gradient dehydration, samples were embedded overnight at 37°C in Pon812 resin (#P1126, SPI Supplies) using acetone as a solvent. Ultrathin sections were stained with 2% uranyl acetate and lead citrate solution, then examined under a Philips CM 120 electron microscope, and ultrastructural images were captured. The thickness of the GBM and the number of podocyte foot processes were analyzed using ImageJ software. Structural changes in podocytes, particularly foot process fusion and cytoskeletal damage, were systematically assessed.

### 2.26 Statistical analysis

All statistical analyses were performed using GraphPad Prism 9.5 (GraphPad Software). Differences among multiple experimental groups were assessed using a one‐way analysis of variance, followed by Tukey's multiple comparisons test. For comparisons between two groups, a two‐tailed Student's *t*‐test was applied. Data are presented as mean ± SD from at least three biologically independent experiments. A *p*‐value <0.05 was considered statistically significant. The significance levels were indicated as follows: *p < 0.05, **p < 0.01, ***p < 0.001.

## 3 RESULTS

### 3.1 Transcriptomic analysis identifies NFATc3 as a key gene in Ang II‐induced podocyte injury

In this study, we successfully established a mouse model of MN and isolated podocytes using magnetic separation. WB analysis was performed to detect the expression levels of podocyte markers nephrin, WT1, and synaptopodin. The results showed a significant decrease in the expression of these markers in the MN model group compared to the control group, indicating the occurrence of podocyte injury (Figure S1).

**FIGURE 1:**
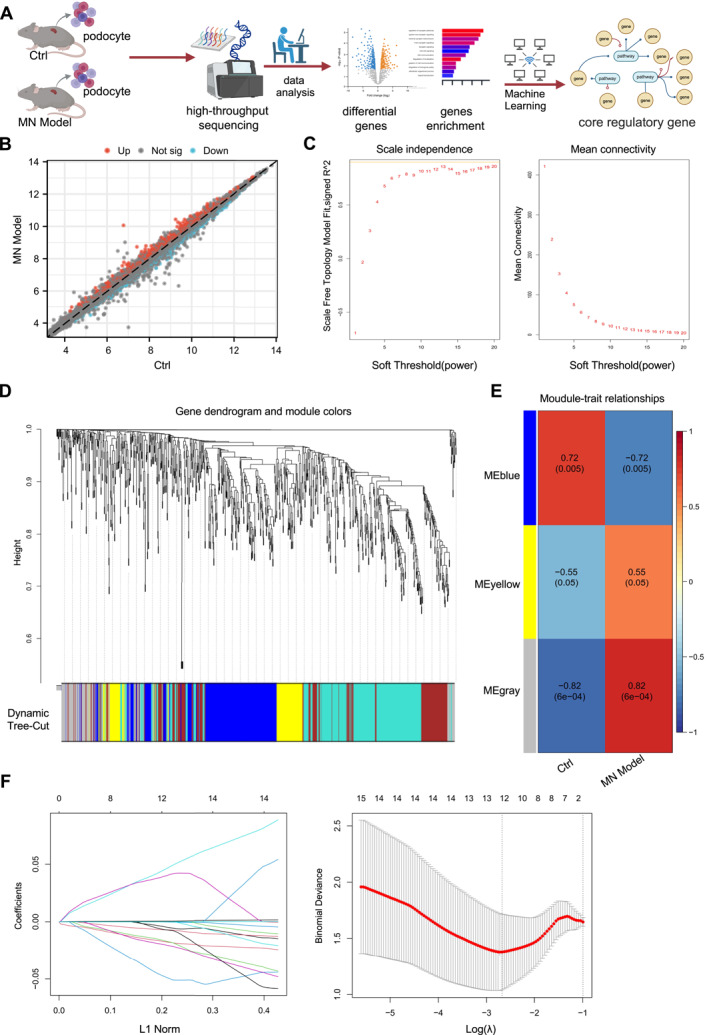
WGCNA and machine learning identify key targets influencing Ang II‐induced podocyte injury. (A) Experimental flowchart of RNA‐seq and machine learning screening of MN model core regulatory genes in podocyte injury; (B) volcano plot analysis of differentially expressed genes in control group and MN model group podocytes; (C) scale independence, mean connectivity, and the selection of the optimal soft‐thresholding power (*β* = 13) to satisfy the scale‐free topology criterion; (D) cluster dendrogram of co‐expression network modules; (E) correlation between gene modules obtained by clustering and MN model; (F) distribution of LASSO regression coefficients (left) and the selection of the optimal lambda value (right) for the LASSO model. Sample size: *n* = 8 per group. LASSO, least absolute shrinkage and selection operator; MN, membranous nephropathy; WGCNA, weighted gene co‐expression network analysis.

Subsequently, RNA‐seq was conducted on podocytes from the two groups using the Illumina HiSeq 2500 platform. Core regulatory genes were identified through computational screening (Figure 1A). Differential expression analysis was performed using the “limma” package in R, revealing a total of 973 significantly DEGs between the MN model and control groups, including 531 upregulated and 442 downregulated genes (Figure 1B, Table S5).

GO enrichment analysis indicated that these DEGs were predominantly associated with BP, such as macromolecule modification, protein modification processes, cytosolic function, and endoplasmic reticulum calcium ion homeostasis (Figure S2A). These processes play crucial roles in intracellular protein folding, calcium ion storage, signal transduction, and cellular stress responses.^35^, ^36^, ^37^ Additionally, KEGG pathway analysis revealed significant enrichment of these DEGs in apoptosis and necroptosis pathways, suggesting that these genes may contribute to podocyte injury by modulating cell death pathways (Figure S2B). These findings imply that podocyte injury in the MN model may be closely associated with ECM remodeling, apoptosis regulation, and calcium homeostasis.

To further identify key regulatory genes, we performed WGCNA based on differentially expressed mRNA data. Using R software, we calculated the optimal soft threshold β for network construction and determined that the best β value satisfying the scale‐free network property was 13 (Figure 1C). Based on this threshold, we set the minimum module size to 50 and the module merging threshold to 0.7 to perform dynamic tree cutting (Figure 1D), resulting in the identification of three gene modules: MEyellow, MEblue, and MEgrey.

We then analyzed the correlation between these modules and clinical traits. Among them, the MEgrey module showed the strongest correlation with the “MN model” trait, with a correlation coefficient of 0.82 and a *p*‐value <0.001 (Figure 1E). Although the MEgrey module typically represents genes that do not cluster into specific functional modules, certain disease‐related genes may be assigned to this module due to inconspicuous expression patterns or lack of significant clustering. These genes may still be functionally relevant to the disease when analyzed independently.^38^ To further refine the selection, we focused on genes within the MEgrey module that exhibited the highest correlation. We employed regression analysis models to fit module genes, eliminating redundant features. The glmnet function was utilized to perform LASSO regression modeling in a binary classification format, extracting sample categories as response variables through regular expressions. The model was evaluated by plotting the model object and conducting cross‐validation with cv.glmnet to determine the optimal lambda value (Figure 1F). This process identified 12 key feature genes. To prioritize core feature genes, we constructed a PPI network and assessed the centrality of these genes, revealing that NFATc3 and DUS2 had the highest centrality scores (Figure S2C,D).

Previous studies have demonstrated that DUS2 modulates tRNA modifications, thereby indirectly regulating protein translation and cellular functions. Meanwhile, NFATc3 can be activated via calcium signaling pathways, translocating into the nucleus to regulate genes associated with cellular stress, apoptosis, and immune responses.^39^, ^40^, ^41^ This direct involvement in cell damage and stress responses aligns well with the pathological mechanisms of podocyte injury and is consistent with our bioinformatics analysis results. Transcriptomic data further showed that NFATc3 was significantly upregulated in the Ang II‐treated group (Figure S2E). Therefore, we hypothesize that NFATc3 may be a critical gene involved in podocyte injury in the MN model.

### 3.2 NFATc3 as a key regulator in Ang II‐induced podocyte injury: In vitro evidence

**FIGURE 2:**
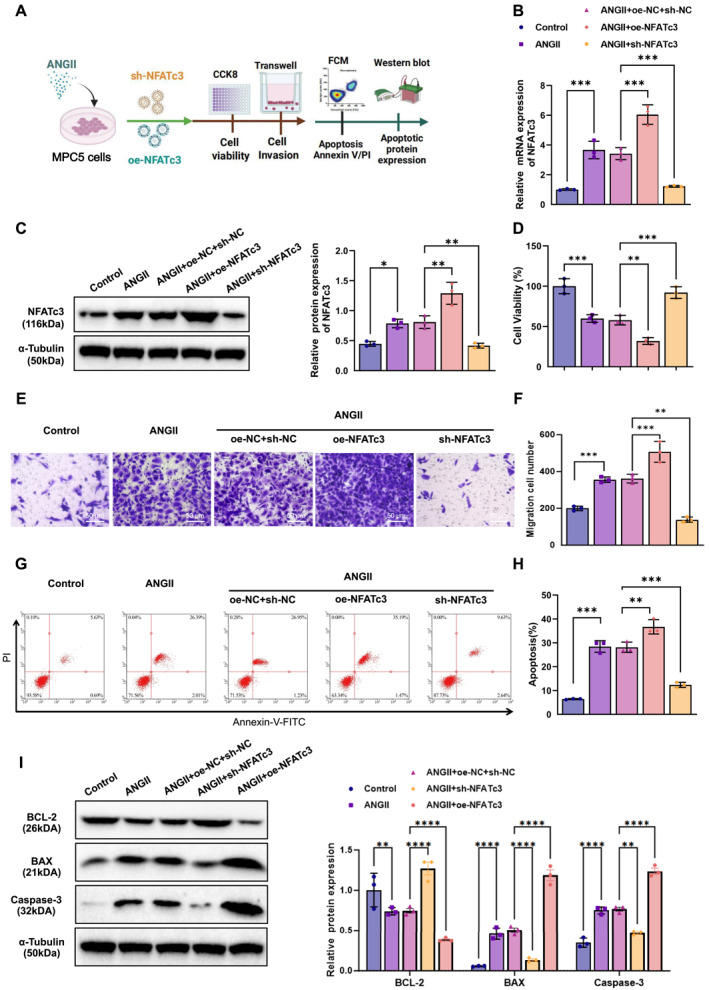
The regulatory role of NFATc3 in Ang II‐induced podocyte injury. (A) Schematic diagram illustrating the experimental workflow of NFATc3's effect on Ang II‐induced podocyte injury; (B, C) qPCR (B) and WB (C) analysis of NFATc3 expression levels across different treatment groups; (D) CCK‐8 assay assessing cell viability; (E) Transwell assay evaluating the effect of NFATc3 on podocyte migration (bar = 50 μm); (F) quantification of migrated cells; (G) annexin V/PI dual staining and flow cytometry analysis of apoptosis rate; (H) statistical analysis of apoptotic cell percentages; (I) WB analysis of apoptosis‐related proteins caspase‐3, BAX, and BCL‐2, along with their relative quantification. All experiments were conducted in triplicate, and data are presented as mean ± SD. NFATc3, nuclear factor of activated T‐cells 3; qPCR, quantitative polymerase chain reaction; SD, standard deviation; WB, Western blot. *p < 0.05, **p < 0.01, ***p < 0.001.

To assess the role of NFATc3 in Ang II‐induced podocyte injury, we modulated NFATc3 expression in podocytes via lentiviral transduction and evaluated cellular behavior using CCK‐8, migration assays, and flow cytometry (Figure 2A). The efficiency of NFATc3 overexpression and silencing was confirmed via qPCR and WB. qPCR results demonstrated a significant upregulation of NFATc3 mRNA levels upon transfection with the NFATc3 overexpression plasmid (oe‐NFATc3), whereas transfection with NFATc3 shRNA (sh‐NFATc3) resulted in a marked decrease in NFATc3 expression. WB analysis corroborated these findings, showing a significant increase in NFATc3 protein levels in the overexpression group and a significant reduction in the silencing group (Figure S3A,B), confirming the successful modulation of NFATc3 expression in podocytes.

Further, qPCR and WB analyses revealed that Ang II treatment induced a significant upregulation of NFATc3 in podocytes. Overexpression of NFATc3 further enhanced its expression, whereas silencing significantly reduced its levels (Figure 2B,C). CCK‐8 assays demonstrated that NFATc3 overexpression exacerbated Ang II‐induced podocyte injury, as indicated by reduced cell viability. Conversely, NFATc3 silencing partially alleviated this damage, leading to improved cell viability (Figure 2D). Transwell assays showed that Ang II treatment significantly enhanced podocyte migration by activating NFATc3. NFATc3 overexpression markedly promoted podocyte migration, while NFATc3 silencing significantly reduced the migratory capacity (Figure 2E,F). Flow cytometry analysis using annexin V/PI staining revealed that NFATc3 overexpression significantly increased apoptotic cell populations, whereas NFATc3 silencing reduced apoptosis (Figure 2G,H). WB analysis of apoptosis‐related proteins demonstrated that caspase‐3 and BAX were significantly upregulated in the NFATc3 overexpression group, whereas BCL‐2 was downregulated. In contrast, NFATc3 silencing led to opposite effects (Figure 2I).

These findings indicate that NFATc3 promotes apoptosis while inhibiting podocyte proliferation and migration, thereby exacerbating Ang II‐induced podocyte injury.

### 3.3 NFATc3 nuclear translocation promotes LRRC55 expression and regulates ECM remodeling in podocytes

Our previous findings indicate that NFATc3 exacerbates podocyte injury, suggesting that it may influence podocyte function by regulating downstream molecules. Previous studies have shown that LRRC55 is a critical regulatory protein of the BK channel and plays a role in ECM remodeling and dysfunction in podocytes. Therefore, investigating whether NFATc3 modulates LRRC55 expression and activity, thereby aggravating ECM remodeling and cellular damage, will provide new insights into the role of NFATc3 in podocyte pathology.

**FIGURE 3:**
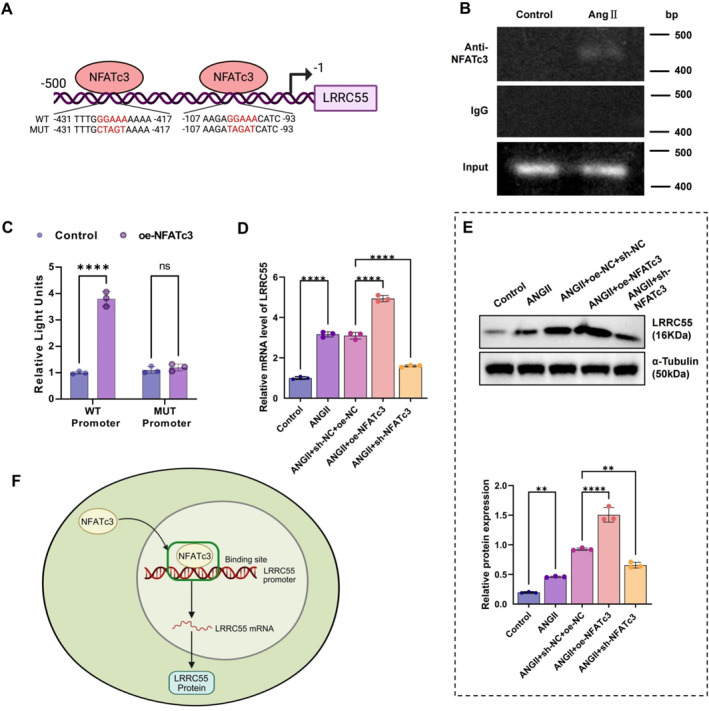
NFATc3 regulates LRRC55 promoter binding and expression. (A) Schematic diagram of NFATc3 binding sites in the LRRC55 promoter DNA sequence, WT and MUT LRRC55 promoter‐luciferase reporter plasmids. (B) ChIP analysis of NFATc3 binding to the LRRC55 promoter in podocytes treated with Ang II (1 μM) for 24 h (GAPDH used as input control in ChIP experiments). (C) NFATc3 overexpression promotes the expression of the luciferase reporter gene containing NFATc3 binding sites; site‐specific mutations affect its activity. (D, E) qPCR and Western blot analysis to detect the regulation of LRRC55 by overexpressed NFATc3 at the mRNA and protein levels. (F) Schematic illustration depicting NFATc3 nuclear translocation and its regulation of LRRC55 expression. All experiments were performed in triplicate, and data are presented as mean ± SD. Statistical significance: ChIP, chromatin immunoprecipitation; LRRC55, leucine‐rich repeat‐containing 55; MUT, mutant; NFATc3, nuclear factor of activated T‐cells 3; qPCR, quantitative polymerase chain reaction; SD, standard deviation; WT, wild type. **p < 0.01, ****p < 0.0001.

We identified two NFATc3‐binding sites within the LRRC55 promoter sequence (Figure 3A). ChIP assays demonstrated that NFATc3 binds to the LRRC55 promoter in podocytes, with this interaction being enhanced following Ang II treatment (Figure 3B). Overexpression of NFATc3 significantly increased the expression of a luciferase reporter construct containing the LRRC55 promoter binding sites, whereas site‐directed mutations abolished this upregulation (Figure 3C). Furthermore, NFATc3 overexpression upregulated endogenous LRRC55 expression at both the mRNA and protein levels in podocytes, whereas NFATc3 knockdown suppressed Ang II‐induced LRRC55 expression (Figure 3D,E). These findings suggest that NFATc3 regulates LRRC55 expression in Ang II‐treated podocytes (Figure 3F) and may play a crucial role in ECM remodeling.

**FIGURE 4:**
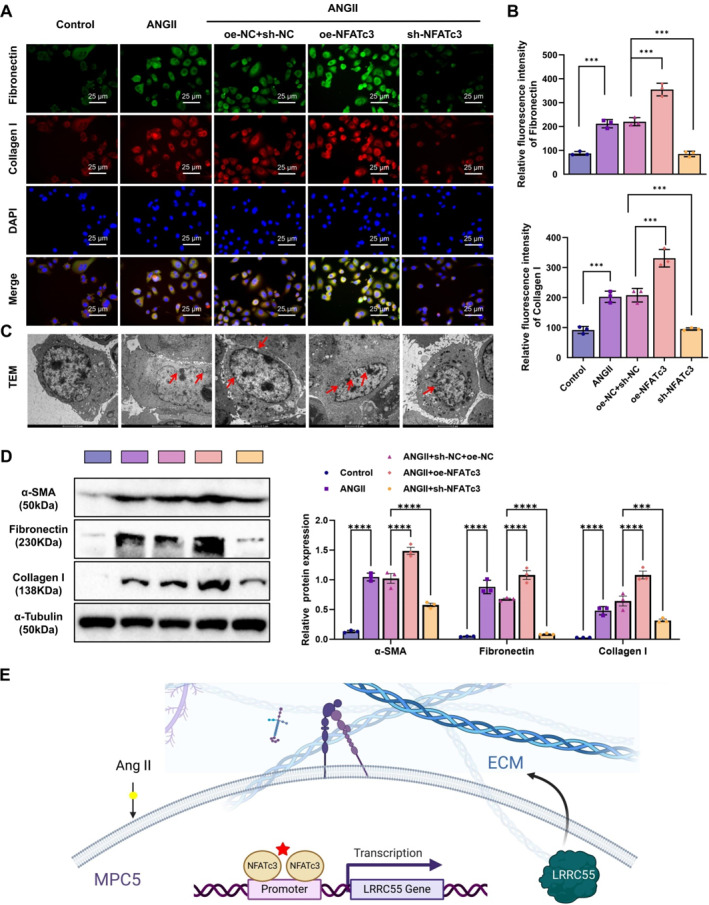
The role of NFATc3 in ECM remodeling in podocytes. (A, B) Immunofluorescence analysis of fibronectin and collagen I expression and distribution (bar = 25 μm); (C) TEM observation of podocyte ultrastructural changes, particularly foot process effacement and cytoskeletal alterations (bar = 2 μm); (D) WB analysis of fibrosis‐related and ECM‐associated protein expression levels; (E) schematic representation of NFATc3‐mediated LRRC55 regulation, exacerbating ECM remodeling. Experiments were performed in triplicate, and data are presented as mean ± SD. ECM, extracellular matrix; LRRC55, leucine‐rich repeat‐containing 55; NFATc3, nuclear factor of activated T‐cells 3; SD, standard deviation; TEM, transmission electron microscopy; WB, Western blot. ***p < 0.001, ****p < 0.0001.

To further investigate ECM remodeling, we analyzed the expression and distribution of ECM‐related proteins, fibronectin and collagen I, using immunofluorescence staining. The results showed that Ang II treatment significantly increased the expression of fibronectin and collagen I in podocytes, with the most pronounced accumulation observed in the NFATc3 overexpression group. Conversely, their expression was relatively lower in the NFATc3 knockdown group (Figure 4A,B). These findings suggest that NFATc3 nuclear translocation may exacerbate podocyte fibrosis by promoting ECM component deposition. WB analysis further confirmed that Ang II‐treated podocytes exhibited increased α‐SMA expression and collagen deposition, indicative of enhanced fibrosis and ECM remodeling. NFATc3 overexpression further amplified fibrotic marker accumulation, whereas NFATc3 knockdown significantly mitigated these effects, reinforcing NFATc3's role in ECM remodeling (Figure 4D). These findings indicate that NFATc3, through the regulation of LRRC55 expression, contributes to excessive ECM accumulation and altered distribution, thereby exacerbating podocyte injury and fibrosis.

To gain deeper insights into podocyte structural changes during ECM remodeling, we employed TEM to examine podocyte ultrastructure, focusing on foot process effacement and cytoskeletal alterations. TEM analysis revealed that Ang II‐treated podocytes exhibited severe foot process effacement and cytoskeletal disorganization, with more pronounced abnormalities in the NFATc3 overexpression group. In contrast, NFATc3 knockdown alleviated these structural defects to some extent (Figure 4C).

Collectively, these observations suggest that NFATc3 exacerbates ECM remodeling and structural disruption by promoting LRRC55 expression and activation, further intensifying Ang II‐induced podocyte injury and fibrosis (Figure 4E).

### 3.4 Upregulated LRRC55 promotes BK channel activation and exacerbates podocyte injury

**FIGURE 5:**
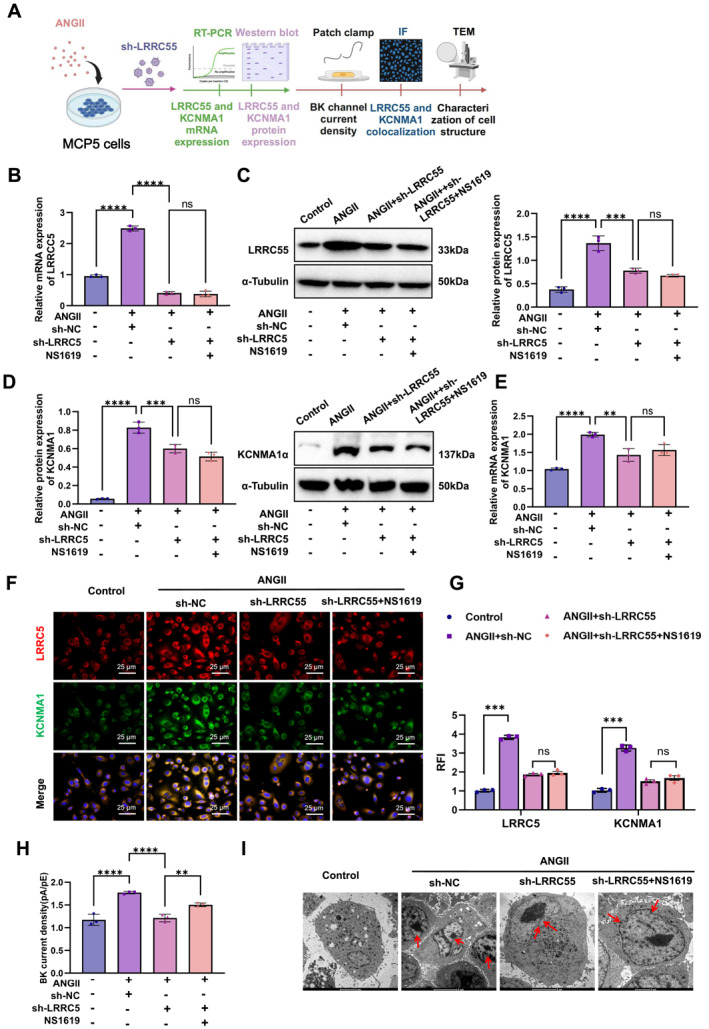
Upregulation of LRRC55 exacerbates podocyte injury by modulating the BK channel. (A) Schematic diagram of the experimental workflow illustrating the treatment conditions for podocytes; (B, C) qPCR and WB analysis of LRRC55 expression in different groups; (D, E) qPCR and WB analysis of BK channel α‐subunit (KCNMA1) expression levels; (F, G) immunofluorescence analysis showing colocalization of LRRC55 and the BK channel α‐subunit on the cell membrane (bar = 25 μm); (H) patch‐clamp analysis of BK channel current density in different groups; (I) TEM imaging of podocyte ultrastructure, highlighting foot process effacement and cytoskeletal alterations (bar = 2 μm). All experiments were performed in triplicate, and data are presented as mean ± SD. BK, big potassium; LRRC55, leucine‐rich repeat‐containing 55; qPCR, quantitative polymerase chain reaction; SD, standard deviation; TEM, transmission electron microscopy; WB, Western blot. **p < 0.01, ***p < 0.001, ****p < 0.0001.

To investigate whether LRRC55 upregulation promotes BK channel activation and aggravates Ang II‐induced podocyte injury, we conducted experiments using podocytes subjected to different treatments (Figure 5A). Furthermore, the expression of the BK channel alpha subunit (KCNMA1 gene) was assessed through qPCR and WB analysis. The results revealed a significant increase in both KCNMA1 mRNA and protein expression in the Ang II‐treated group. Conversely, in the LRRC55 knockdown group, the expression of KCNMA1 was significantly reduced. Notably, the addition of the BK channel activator NS1619 had no significant impact on the protein and mRNA levels of KCNMA1 (Figure 5B,C). These findings suggest that Ang II‐induced LRRC55 upregulation may contribute to podocyte injury.

Furthermore, qPCR and WB assays were used to assess the expression of the BK channel α‐subunit (encoded by KCNMA1). The results demonstrated that KCNMA1 mRNA and protein levels were significantly elevated in the Ang II‐treated group, whereas KCNMA1 expression was markedly reduced in the LRRC55 knockdown group. The addition of NS1619 partially restored KCNMA1 protein expression but had no significant effect on KCNMA1 mRNA levels (Figure 5D,E). These findings indicate that LRRC55 upregulation may enhance KCNMA1 expression, thereby promoting BK channel activation and exacerbating podocyte injury.

Immunofluorescence analysis further revealed a significant increase in the colocalization of LRRC55 and the BK channel α‐subunit at the plasma membrane (Figure 5F,G), suggesting that LRRC55 may enhance potassium ion transport by activating BK channels. Additionally, whole‐cell patch‐clamp recordings demonstrated that BK channel current density was significantly increased in the Ang II‐treated group but markedly reduced in the LRRC55 knockdown group. Notably, the addition of NS1619 partially restored BK channel current density in the LRRC55 knockdown group (Figure 5H). These results suggest that LRRC55 plays a critical role in regulating BK channel activity and contributing to podocyte injury.

To evaluate the structural impact of LRRC55 on podocytes, TEM was performed. The results showed that Ang II treatment significantly exacerbated foot process effacement and cytoskeletal disorganization. These pathological changes were alleviated in the LRRC55 knockdown group but reappeared in the NS1619‐treated group (Figure 5I).

In conclusion, LRRC55 upregulation enhances KCNMA1 expression, activates BK channels, and exacerbates Ang II‐induced podocyte injury and ECM remodeling.

### 3.5 In vivo validation of the NFATc3/LRRC55/BK axis in ECM remodeling and podocyte injury in MN

**FIGURE 6:**
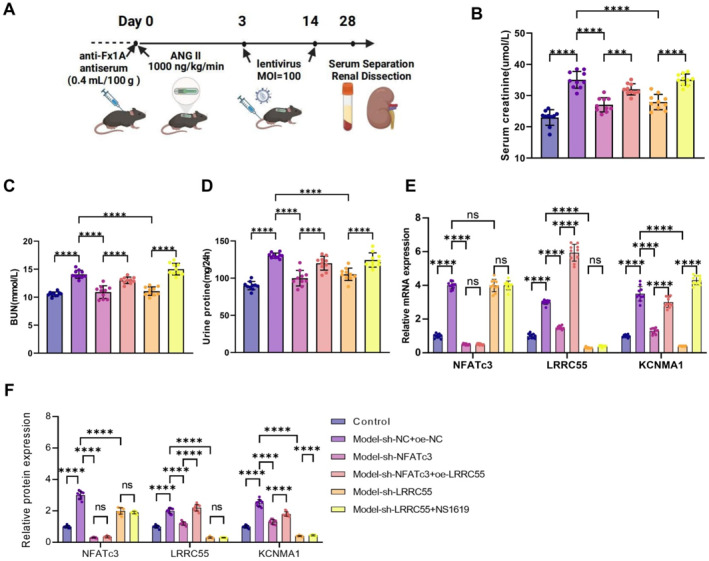
Role of the NFATc3/LRRC55/BK channel axis in the MN model. (A) Schematic representation of the in vivo experimental procedure, illustrating the construction and treatment of the MN mouse model; (B–D) quantification of Scr (B), BUN (C), and 24‐h urinary protein levels (D); (E, F) qPCR (E) and WB (F) analysis of NFATc3, LRRC55, and KCNMA1 mRNA and protein expression in renal tissues. Each group consisted of 10 mice, and data are presented as mean ± SD. BK, big potassium; BUN, blood urea nitrogen; LRRC55, leucine‐rich repeat‐containing 55; MN, membranous nephropathy; NFATc3, nuclear factor of activated T‐cells 3; qPCR, quantitative polymerase chain reaction; Scr, serum creatinine; SD, standard deviation; WB, Western blot. ****p < 0.00.

To validate the role of the NFATc3/LRRC55/BK channel axis in MN, we established an MN mouse model via a single injection of anti‐Fx1A antiserum combined with continuous infusion of angiotensin II (Ang II) using ALZET osmotic pumps. Renal function parameters were subsequently evaluated (Figure 6A). The results showed that mice in the model group exhibited significantly elevated levels of Scr and BUN, along with a marked increase in 24‐h urinary protein excretion, indicating impaired glomerular filtration function. Compared with the model group, renal function parameters were significantly improved in the NFATc3 knockdown group (model + sh‐NFATc3), whereas renal injury was further exacerbated in the LRRC55 overexpression group (model + sh‐NFATc3 + oe‐LRRC55). Notably, administration of the BK channel agonist NS1619 led to even more severe renal dysfunction (Figure 6B–D).

To further explore the regulatory effects of the NFATc3/LRRC55/BK channel axis on gene expression, we performed qPCR and WB analyses to assess NFATc3, LRRC55, and KCNMA1 (the α‐subunit of the BK channel) expression levels in renal tissues. qPCR results demonstrated a significant upregulation of NFATc3, LRRC55, and KCNMA1 mRNA levels in the model group, whereas their expression was markedly reduced in the NFATc3‐knockdown group. WB analysis corroborated these findings, showing increased LRRC55 and KCNMA1 protein levels in the LRRC55‐overexpression group and significant downregulation in the LRRC55‐knockdown group (Figure 6E,F). These results suggest that NFATc3 modulates LRRC55 expression, thereby influencing BK channel activity and contributing to glomerular injury.

**FIGURE 7:**
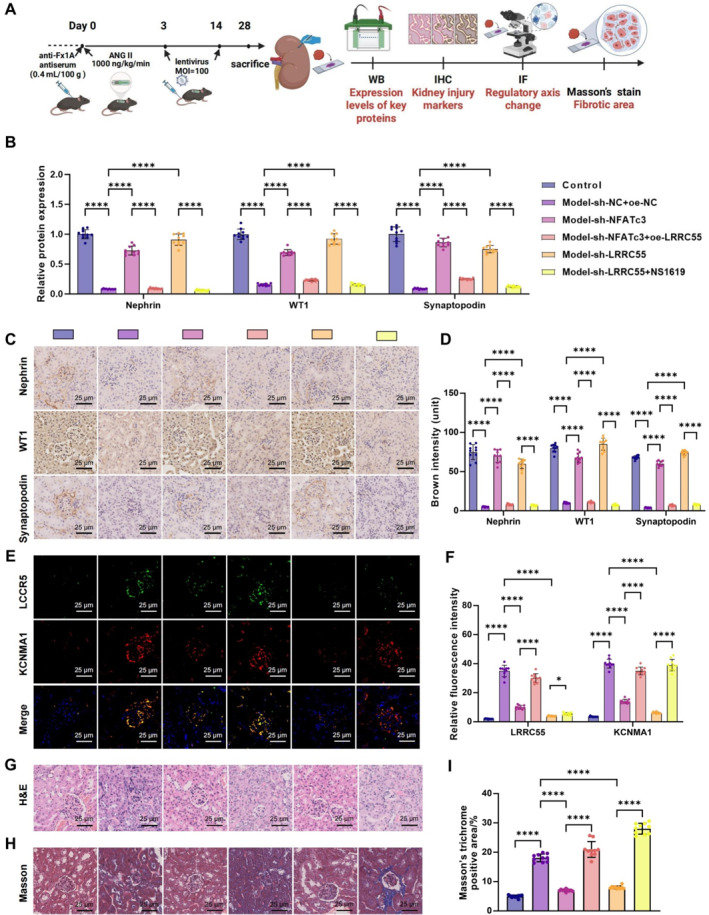
Analysis of the NFATc3/LRRC55/BK channel axis in podocyte injury and renal pathology. (A) Experimental workflow for assessing renal injury in different treatment groups; (B) WB analysis of nephrin, WT1, and synaptopodin expression; (C, D) immunohistochemical detection of nephrin, WT1, and synaptopodin (scale bar = 25 μm); (E, F) immunofluorescence staining showing the expression and colocalization of LRRC55 and the BK channel α‐subunit in glomeruli (scale bar = 25 μm); (G) H&E staining illustrating GBM thickening and tubular atrophy (scale bar = 25 μm); (H, I) Masson's trichrome staining indicating the degree of renal fibrosis (scale bar = 25 μm). Data are presented as mean ± SD for *n* = 10 mice per group; BK, big potassium; GBM, glomerular basement membrane; H&E, hematoxylin and eosin; LRRC55, leucine‐rich repeat‐containing 55; NFATc3, nuclear factor of activated T‐cells 3; SD, standard deviation; WB, Western blot. ****p < 0.0001.

Histopathological examination of renal tissues from different experimental groups further assessed the extent of kidney damage (Figure 7A). WB and immunohistochemical analyses revealed significant downregulation of podocyte markers nephrin, WT1, and synaptopodin in the model group. In contrast, NFATc3 knockdown restored the expression of these markers, whereas LRRC55 overexpression further reduced their levels, indicating exacerbated podocyte injury (Figure 7B–D). Immunofluorescence staining revealed a marked increase in LRRC55 and BK channel α‐subunit expression in glomeruli, particularly in the LRRC55‐overexpression group, where their colocalization was more prominent. This finding suggests that LRRC55 upregulation may activate the BK channel, thereby exacerbating glomerular damage (Figure 7E,F).

To evaluate renal histopathological changes, we performed H&E and Masson's trichrome staining. H&E staining demonstrated thickening of the GBM, significant podocyte injury, and varying degrees of tubular atrophy in the Ang II‐treated group (Figure 7G). Masson's staining revealed extensive renal fibrosis, which was further aggravated in the LRRC55‐overexpression group, whereas fibrosis was significantly alleviated in the NFATc3‐knockdown group (Figure 7H,I). These findings indicate that the NFATc3/LRRC55/BK channel axis plays a critical role in podocyte ECM remodeling and renal fibrosis.

Collectively, our results suggest that the NFATc3/LRRC55/BK channel axis regulates podocyte structure and function, thereby promoting Ang II‐induced MN progression.

## 4 DISCUSSION

This study elucidates the pivotal role of NFATc3 in Ang II‐induced podocyte injury in MN. As a transcription factor, NFATc3 is known to be involved in immune responses, cell proliferation, and migration. Previous studies have demonstrated its role in regulating immune and inflammatory responses in various disease models, particularly in kidney diseases, where its aberrant expression has been linked to tubular injury, renal fibrosis, and loss of kidney function.^42^, ^43^, ^44^ Although some studies have explored the involvement of NFATc3 in renal injury, our findings clearly demonstrate that NFATc3 exacerbates Ang II‐induced podocyte damage by regulating the function of LRRC55 and BK channels. This provides novel insights into the molecular mechanisms underlying the pathogenesis of MN.

The roles of LRRC55 and BK channels in renal physiology are intricate and have been a focus of extensive research. LRRC55, an ion channel regulatory protein, has been shown to interact with BK channels to modulate ion homeostasis, thereby influencing cellular function and injury responses.^28^, ^45^, ^46^ Consistent with previous findings, this study demonstrates that LRRC55 exacerbates Ang II‐induced podocyte injury by regulating BK channel function. However, although the literature has primarily focused on LRRC55 in the nervous and muscular systems, its role in renal injury remains underexplored. Our findings highlight the significance of the LRRC55/BK channel axis in podocyte injury, further advancing our understanding of this channel system in kidney pathophysiology.

This study demonstrates that NFATc3 significantly influences ECM remodeling in podocytes by regulating the LRRC55/BK channel axis. Aberrant ECM accumulation is a hallmark of MN, where excessive ECM deposition can lead to renal fibrosis and functional deterioration. Previous studies have shown that podocyte injury promotes renal fibrosis by inducing abnormal ECM protein expression and accumulation, with NFATc3 activation identified as a critical regulatory factor in this process.^22^, ^47^, ^48^, ^49^ Our findings further reveal that NFATc3 modulates ECM composition via the LRRC55/BK channel axis, exacerbating renal injury. This discovery provides novel insights into the link between podocyte damage and fibrosis. Unlike conventional research approaches, our study integrates NFATc3, ion channels, and ECM remodeling, unveiling a previously unrecognized molecular mechanism.

Ang II is a key pathogenic factor in MN, known to enhance podocyte apoptosis, migration, and ECM remodeling by activating multiple renal signaling pathways.^50^, ^51^ In this study, we established an Ang II‐induced podocyte injury model to investigate the role of the NFATc3/LRRC55/BK channel axis. Consistent with previous reports, Ang II induced podocyte damage and promoted ECM remodeling and cellular migration through NFATc3‐mediated signaling. Although previous studies have reported on the role of the NFATc3/LRRC55/BK channel axis, the study by Hu et al.^28^ primarily focused on the direct function of LRRC55 and BK channels and their involvement in apoptosis. In contrast, our study further elucidates the NFATc3‐mediated regulation of LRRC55 in the context of ECM remodeling and cytoskeletal alterations in podocytes—mechanisms that have not been thoroughly explored previously. However, for the first time, we demonstrate that NFATc3 exerts its effects via the LRRC55/BK channel axis. This novel finding enhances our understanding of Ang II‐induced renal injury and offers new potential targets for therapeutic intervention in MN.

Podocyte injury plays a crucial role in the progression of kidney diseases, particularly in MN and other glomerular disorders. Damage to podocytes not only disrupts the glomerular filtration barrier but also triggers fibrotic responses through multiple signaling pathways. Previous studies have demonstrated that podocyte injury activates various cytokines and transcription factors, leading to excessive ECM accumulation and fibrosis.^12^, ^52^, ^53^ The findings of this study further confirm the critical role of podocyte injury in renal fibrosis. Specifically, we identified that the NFATc3/LRRC55/BK channel axis exacerbates podocyte damage and accelerates fibrosis. These findings enhance our understanding of the intricate relationship between podocyte injury and renal fibrosis, providing a potential molecular target for therapeutic intervention.

Current treatment strategies for MN primarily focus on modulating immune responses and reducing proteinuria; however, no significant breakthroughs have been achieved in targeting podocyte injury and fibrosis. This study proposes the NFATc3/LRRC55/BK channel axis as a novel therapeutic target for MN, offering new insights into renal injury intervention. Although previous research has explored the role of ion channels in kidney diseases, our study is the first to establish a direct link between NFATc3 and ion channels in this context. This novel insight paves the way for future molecular‐targeted therapies for MN.

By elucidating the mechanistic role of the NFATc3/LRRC55/BK channel axis in Ang II‐induced MN, this study provides a new regulatory mechanism for podocyte injury, broadening our understanding of MN pathogenesis. Scientifically, our integrative approach—combining transcription factors, ion channels, and ECM remodeling—identifies novel molecular targets and therapeutic strategies. Clinically, the NFATc3/LRRC55/BK channel axis may serve as a promising therapeutic target for MN, particularly in patients unresponsive to conventional treatments.

Despite these significant findings, our study does not explore the role of the NFATc3/LRRC55/BK channel axis in other types of nephropathy, necessitating further research to validate its broader implications. Additionally, although in vivo models provided strong evidence supporting our conclusions, extensive clinical trials are required for translational applications. Future research should focus on developing specific inhibitors targeting NFATc3 or the BK channel and evaluating their potential clinical utility.

## 5 CONCLUSION

This study demonstrates that NFATc3 exacerbates Ang II‐induced podocyte injury and the progression of MN by upregulating LRRC55 expression and activating the BK ion channel. Through both in vitro and in vivo experiments, we confirmed the critical role of the NFATc3/LRRC55/BK channel axis in ECM remodeling and podocyte injury. Overexpression of LRRC55 not only significantly enhanced BK channel activity in podocytes but also contributed to excessive ECM accumulation, ultimately leading to glomerular structural damage and renal dysfunction. In contrast, silencing NFATc3 effectively mitigated these pathological changes, suggesting its potential as a therapeutic target for MN.

**FIGURE 8:**
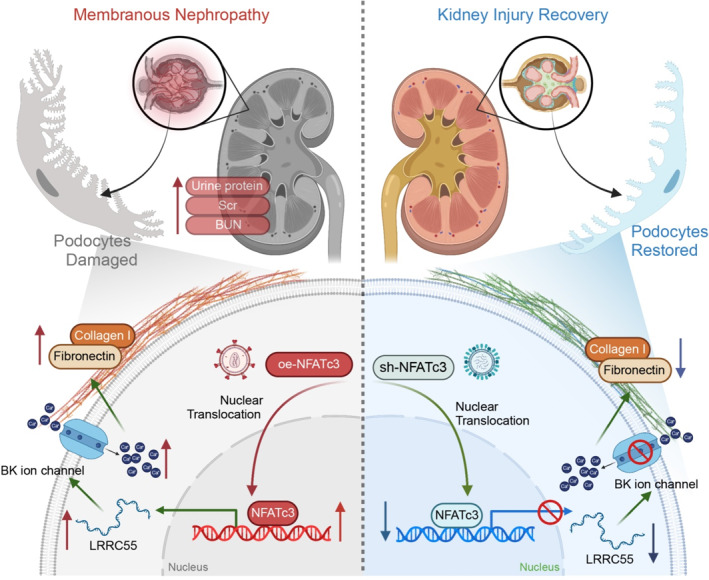
Molecular mechanism of NFATc3/LRRC55‐mediated ECM remodeling via the BK ion channel in aggravating Ang II‐induced podocyte injury in MN. BK, big potassium; ECM, extracellular matrix; LRRC55, leucine‐rich repeat‐containing 55; MN, membranous nephropathy; NFATc3, nuclear factor of activated T‐cells 3.

Our findings elucidate the pivotal role of the NFATc3/LRRC55/BK channel axis in podocyte injury and glomerular pathology, providing novel insights into the molecular mechanisms underlying MN. The regulatory function of NFATc3 and its downstream signaling pathways presents a promising avenue for MN therapy, with LRRC55 and the BK channel emerging as potential therapeutic targets. Future drug development targeting these molecules may offer improved prognostic outcomes for MN patients (Figure 8).

## AUTHOR CONTRIBUTIONS

Yaling Guo and Weidong Chen conceived and designed the study. Jingliang Min, Baochao Chang, and Lei Liu performed the experiments. Yaling Guo and Jiqiang Zhang analyzed the data. Yaling Guo and Weidong Chen wrote the manuscript. All authors reviewed and approved the final version of the manuscript.

## CONFLICT OF INTEREST STATEMENT

The authors declare no conflicts of interest.

## ETHICS STATEMENT

All animal experiments were approved by the Animal Ethics Committee of the First Affiliated Hospital of Bengbu Medical University (No. [2024]327).

## Supporting information

Figures S1–S3

Tables S1–S4

Table S5

## Data Availability

All data can be provided as needed.
