# Supplementary material for: Modulation of podocyte extracellular matrix remodeling in membranous nephropathy by the NFATc3/LRRC55/BK channel pathway
Source: J Cell Commun Signal. 2025 Jun 13;19(2):e70022. doi: 10.1002/ccs3.70022 (PMC12165835; doi:10.1002/ccs3.70022)
Supplement: Supplementary file 1 — Figures S1–S3 [file CCS3-19-e70022-s002.docx]

**
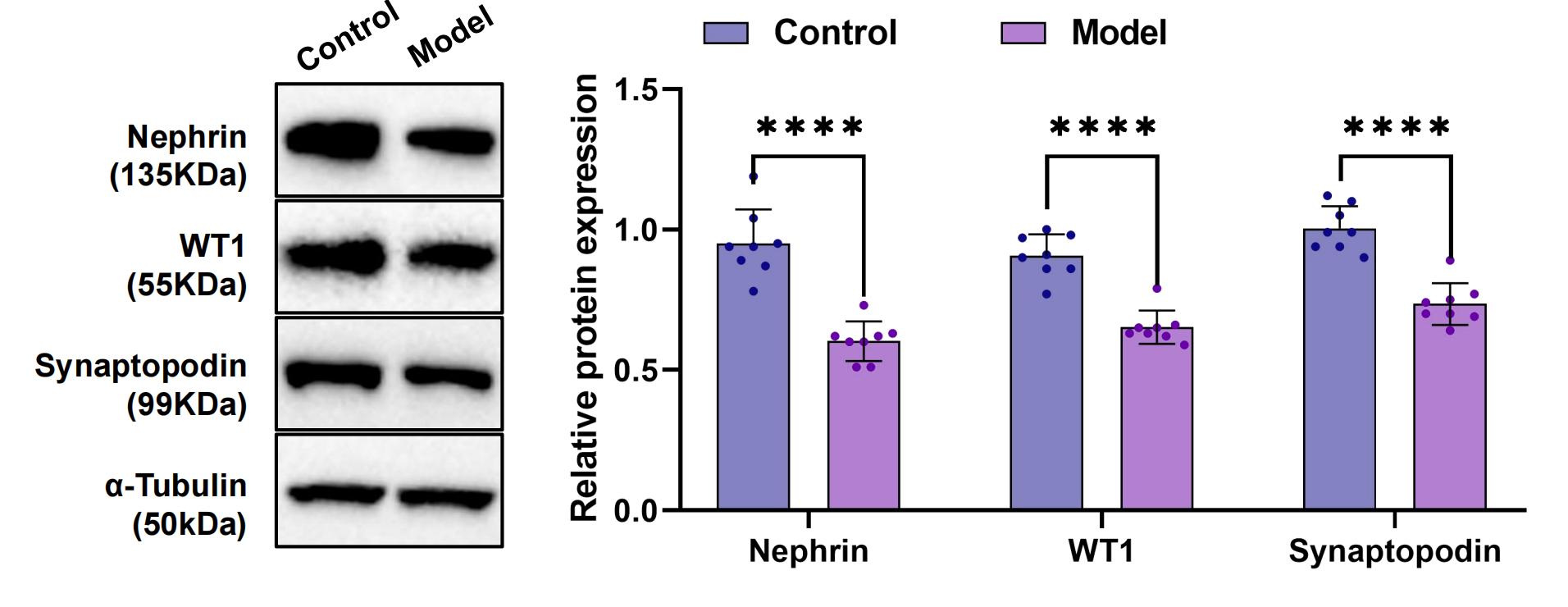
**

**Figure S1. WB Analysis of Podocyte Markers Nephrin, WT1, and Synaptopodin Expression Levels.**

**
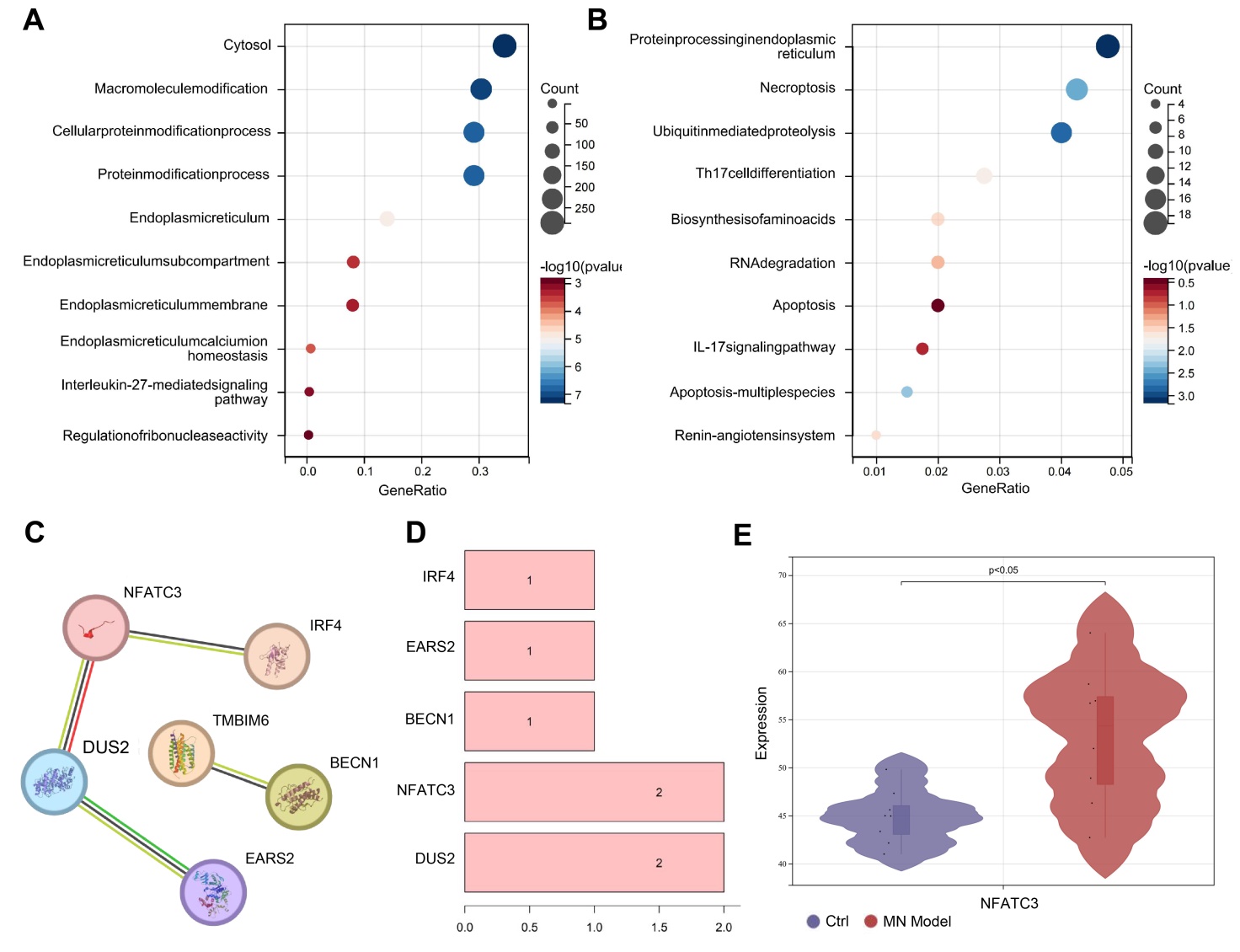
**

**Figure S2. Differential Gene Enrichment Analysis and Identification of Key Regulatory Factors.**

Note: (A) GO enrichment analysis of differentially expressed genes; (B) KEGG enrichment analysis; (C) PPI network of key regulatory factors; (D) Statistical analysis of PPI network node connectivity, with the x-axis representing the number of connections—higher values indicate greater centrality, showing the top five ranked genes; (E) Transcriptomic expression levels of NFATc3.

**
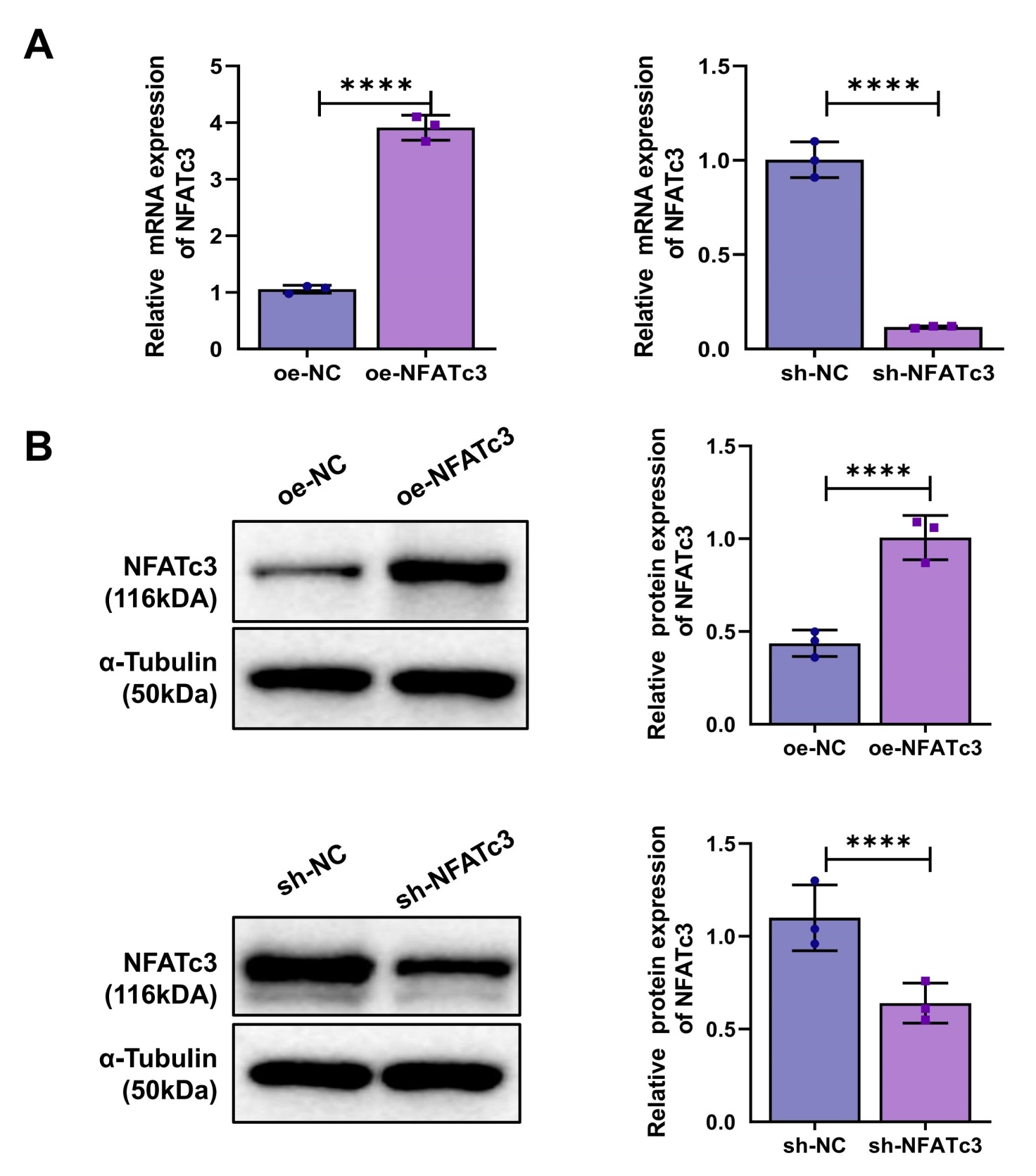
**

**Figure S3. Validation of NFATc3 Overexpression and Silencing.**

Note: (A) qPCR analysis of NFATc3 mRNA expression levels in the overexpression and silencing groups; (B) WB validation of NFATc3 protein expression, confirming the effectiveness of overexpression and silencing. Experiments were conducted in triplicate, and data are presented as mean ± SD. **p* < 0.05, ***p* < 0.01, ****p* < 0.001.
