# Supplementary material for: Modulation of podocyte extracellular matrix remodeling in membranous nephropathy by the NFATc3/LRRC55/BK channel pathway
Source: J Cell Commun Signal. 2025 Jun 13;19(2):e70022. doi: 10.1002/ccs3.70022 (PMC12165835; doi:10.1002/ccs3.70022)
Supplement: Supplementary file 2 — Tables S1–S4 [file CCS3-19-e70022-s001.docx]

**Table S1. Primer Sequences for Lentiviral Transfection.**

| **Name** | **Sequence (5'-3')** |
| --- | --- |
| sh-NFATc3 | GCTCACATTGTCCTTGAAGTT |
| sh-LRRC55 | AGCTCATGGACTGGTACATAT |
| sh-NC | GCAAGCTGACCCTGAAGTTCAT |

**Table S2. RT-qPCR Primer Sequences.**

| **Gene** | **Primer** |
| --- | --- |
| NFATc3 (Mouse) | F 5'- CAGTTGCTCTGTCAATGGAGGC-3' |
|  | R 5'- TTCCTTCAGCCTCCCAATGAGG-3' |
| LRRC55 (Mouse) | F 5'- TGAAGTGGCTGCGGAATCGGAT-3' |
|  | R 5'- GAGGTAATCATCCAGGGTCAGAG-3' |
| KCNMA1 (Mouse) | F 5'-CCTGAAGGACTTTCTGCACAAGG-3' |
|  | R 5'-ACTCCACCTGAGTGAAATGCCG-3' |
| GAPDH (Mouse) | F 5'-CATCACTGCCACCCAGAAGACTG-3' |
|  | R 5'-ATGCCAGTGAGCTTCCCGTTCAG-3' |

Note: F, forward; R, reverse.

**Table S3. Details of Primary Antibodies.**

| **Name** | **Cat.** | **Dilution ratio** | **Manufacturer** |
| --- | --- | --- | --- |
| NFATC3 | PA5-79734 | 0.1-0.5 µg/mL | Thermo Fisher Scientific |
| LRRC55 | ab121412 | 1:100 | Abcam |
| KCNMA1 | APC-151 | 1:100 | Alomone Labs |
| Nephrin | PA5-20330 | 1 µg/mL | Thermo Fisher Scientific |
| WT1 | MA5-32215 | 1:2000 | Thermo Fisher Scientific |
| Synaptopodin | ab259976 | 1:1000 | Abcam |
| α-Tubulin | ab7291 | 1:1000 | Abcam |

**Table S4. Primer Sequences for Site-Directed Mutagenesis.**

| **Name** | **Sequence (5'-3')** |
| --- | --- |
| LRRC55-MUT-1-F | CTCTCTTTGGctagtAAAGAGGCTTCCTGGG |
| LRRC55-MUT-1-R | GAGGACCCCAGGTGA |
| LRRC55-MUT-2-F | GCACCAAGAGtagatATCCAGCAGGCATGG |
| LRRC55-MUT-2-R | TGTGTCTGGGAGGGAG |
